# Unfair offers, unfair offenders? Fairness considerations in incarcerated individuals with and without psychopathy

**DOI:** 10.3389/fnhum.2013.00406

**Published:** 2013-07-26

**Authors:** Sina Radke, Inti A. Brazil, Inge Scheper, Berend H. Bulten, Ellen R. A. de Bruijn

**Affiliations:** ^1^Donders Institute for Brain, Cognition and Behaviour, Radboud University NijmegenNijmegen, Netherlands; ^2^PompestichtingNijmegen, Netherlands; ^3^Donders Institute for Brain, Cognition and Behaviour, Radboud University Nijmegen Medical CentreNijmegen, Netherlands; ^4^Department of Clinical, Health and Neuropsychology, Leiden Institute for Brain and Cognition, Leiden UniversityLeiden, Netherlands

**Keywords:** psychopathy, social decision-making, ultimatum game, fairness, antisocial

## Abstract

Offenders with psychopathy have often committed crimes violating social norms, which may suggest a biased moral reasoning in psychopathy. Yet, as findings on utilitarian decisions remain conflicting, the current study investigated different aspects of fairness considerations in offenders with psychopathy, offenders without psychopathy and healthy individuals (*N* = 18/14/18, respectively). Unfair offers in a modified Ultimatum Game (UG) were paired with different unselected alternatives, thereby establishing the context of a proposal, and made under opposing intentionality constraints (intentional vs. unintentional). As in previous studies, unfair offers were most often rejected when the alternative was fair and when the offer was made intentionally. Importantly, however, offenders with psychopathy demonstrated a similar rejection pattern to that of healthy individuals, i.e., taking the unselected alternative into account. In contrast, delinquents without psychopathy did not adjust their decision behavior to the alternatives to an offer, suggesting stronger impairments in social decision-making. Crucially, the mechanisms and processes underlying rejection decisions might differ, particularly with regard to cognitive vs. emotional competencies. While preserved cognitive perspective-taking could drive seemingly intact decision patterns in psychopathy, emotional empathy is likely to be compromised.

## Introduction

Social deficits are evident in various psychiatric disorders with their expression ranging from withdrawal in e.g., social phobia to antisocial behavior and even social predation as observed in psychopathy. Offenders with psychopathy often show a history of serious violent crimes committed against another person (i.e., murder, rape) and increased recidivism of criminal behavior (D'Silva et al., 2004) that exceeds the relapse rate of offenders without psychopathy by a factor of up to four (Harris et al., 1991; Hemphill et al., 1998).

The initial concept of psychopaths as “moral imbeciles” (Maudsley, 1895) attributed their deviations to a decreased ability for moral reasoning. Indeed, core traits of psychopathy such as manipulative behavior, callousness, and lack of guilt/remorse have been associated with overlooking moral principles for non-moral incentives such as money as well as a negative appreciation of the moral values of fairness and harm prevention (Glenn et al., 2009). In order to target the cognitive component of morality, hypothetical moral dilemmas that assess decisive judgments, e.g., how “appropriate” an action in the given situation is or whether one would execute that action, are frequently used (Greene et al., 2001). Although offenders with psychopathy tend to maximize overall benefit in these scenarios, i.e., demonstrate utilitarian choice patterns (Koenigs et al., 2012), there is no consistent evidence that individuals with psychopathy differ from healthy groups in explicit moral judgments (Blair et al., 1995; Cima et al., 2010; Aharoni et al., 2012). Findings on differences in moral reasoning between incarcerated populations with and without psychopathy are similarly inconsistent (Cima et al., 2010; Koenigs et al., 2012) and a recent meta-analysis reported a negative relation between moral development and recidivism for offenders in general, irrespective of psychopathic traits (Van Vugt et al., 2011). It therefore remains important to compare individuals with psychopathy not only to a healthy, but also to another forensic reference group which has also been convicted for serious offenses that essentially violate social and moral norms.

In contrast to hypothetical scenarios, an association between psychopathic traits and an increased focus on self-interest has been derived from social decision-making paradigms (Rilling et al., 2007; Mokros et al., 2008; Koenigs et al., 2010; Osumi and Ohira, 2010). Economic games, such as the Ultimatum Game (UG; Güth et al., 1982), are frequently used to capture strategies in interpersonal settings that involve weighting self-interest and other-interest. Here, the first player proposes a split of a resource, which can be either accepted or rejected by the second player (responder). Acceptance implements the proposal, but rejection leaves both players with nothing. Instead of “rationally” maximizing their payoff by accepting anything, responders frequently reject unfair offers, which has been attributed to fairness considerations (Güth et al., 1982).

In individuals with psychopathic traits, the observed disregard for fairness norms (Glenn et al., 2009; Aharoni et al., 2011) is mirrored in altered responder behavior in the UG, although the findings remain conflicting. On the one hand, students scoring high on psychopathic traits displayed lower rejection rates of unfair offers, interpreted as favoring self-interest (Osumi and Ohira, 2010). On the other hand, incarcerated patients with psychopathy showed the opposite pattern: individuals with primary psychopathy, i.e., psychopathy with low trait anxiety, rejected more unfair offers relative to individuals with secondary psychopathy or without psychopathy, which the authors relate to deficits in regulating anger and frustration (Koenigs et al., 2010). A recently published study found similar rejection behavior in participants with high and low psychopathic tendencies and suggests different underlying decision mechanisms, i.e., rejection as a reaction to frustration in individuals scoring high on psychopathic traits (Vieira et al., 2013).

As the classic UG assesses outcome-based fairness considerations, i.e., a comparison of outcomes of the self and the other (Radke et al., 2012), without an explicit normative reference point, it remains unresolved which factors underlie the deviations in social decision-making. Along these lines, previous results on psychopathy and UG decisions (Koenigs et al., 2010; Osumi and Ohira, 2010) can only be interpreted on the basis of outcome-driven judgments, but not in terms of social dynamics. In contrast, information derived from context and perceived intentionality guide not only social interactions in our daily lives, but also influence UG decisions (Blount, 1995; Falk et al., 2003, 2008; Güroğlu et al., 2009; Radke et al., 2012).

Interestingly, a recent finding revealed that offenders with psychopathy rate accidents as more morally permissible than delinquents without psychopathy (Young et al., 2012). This inclination suggests that they might weight the intention behind an action greater than its (harmful) outcome, stemming from the deficit of generating an emotional response to the victim's suffering (Young et al., 2012). It remains open, however, in how far this partiality in moral judgments might also apply to imbalanced decision-making. Of note, in laboratory settings, moral judgments are made from a detached perspective as the situation to be judged remains hypothetical, even when probed by a “would you do … in order to … ?” question. Therefore, both the implementation of one's choice and the absence vs. presence of self-interest are important methodological distinctions between the use of hypothetical scenarios and socioeconomic games. Whereas the former usually depict vignettes or actions that do not affect oneself, economic games traditionally involve real, to-be-paid-out stakes and thus outcomes relevant to oneself and one's interaction partner.

Using a modified UG enables us to investigate how social decisions involving fairness considerations are resolved. In this version, information is provided about an unselected alternative, thereby establishing the “context” in which an offer is selected, and about the intentionality of an offer. From a fixed set of two allocations of 10 coins, either the first player (proposer) himself or the computer randomly chooses one. The fixed set allows to manipulate the reference point (“context”) of the proposal (Falk et al., 2003; Güroğlu et al., 2009; Radke et al., 2012), whereas the agency of the proposer constitutes the manipulation of intentionality, i.e., whether the offer was selected by proposers themselves vs. by the computer (Radke et al., 2012). This setup allows to investigate perspective-taking from the side of responders. Here, of particular interest are unfair proposals (8 coins for the proposer and 2 coins for the responder) that are contrasted against either fair, hyperfair, or hyperunfair alternatives, or no alternative at all. Previous findings show that unfair offers are more often rejected when the alternative was fair compared to all other three alternatives (Falk et al., 2003; Güroğlu et al., 2009; Radke et al., 2012), which has also been associated with developmental advances in cognitive perspective-taking abilities (Güroğlu et al., 2009). In adults, a similar rejection pattern was evident for intentional and unintentional decisions (Radke et al., 2012). However, intentionality played a crucial role in the decision process when an unfair treatment was made explicit and salient, i.e., when paired with a fair alternative: These unfair offers were more often rejected when the offer was selected by proposers themselves than when selected by the computer, underlining the importance of punishing intentional social norm violations (Radke et al., 2012).

The current study is the first to investigate social decision-making based on different aspects of fairness considerations and their (social-) cognitive demands (Radke et al., 2012) in a forensic sample. The behavior of offenders with psychopathy was compared to a group of offenders without psychopathy and a group of healthy controls. For healthy individuals, we expected to replicate previous findings on the effects of context referring to the manipulation of alternative offers as in (Güroğlu et al., 2009; Radke et al., 2012) and intentionality (Radke et al., 2012). As these features of social decision-making have not been assessed in our populations of interest until now, it is difficult to predict the rejection patterns in the offender groups.

Based on studies pointing to a relative integrity of cognitive functioning in psychopathy as opposed to non-psychopathy (Morgan and Lilienfeld, 2000; Gao and Raine, 2009; Brazil et al., 2012), one might expect the group with psychopathy to take the context or the intentionality of an offer into account. On the contrary, offenders without psychopathy might show a more impulsive behavioral pattern, not differentiating on the basis of additional information. In sum, we aimed to investigate to what extent the alterations in moral judgments in psychopathy and non-psychopathy translate to decisions with not only moral, but also utilitarian outcomes.

## Materials and methods

### Participants

The offender groups were recruited from the patient population of the Pompestichting Forensic Psychiatric Institute in Nijmegen, The Netherlands^1^. The study was approved by the local medical ethics committee and in accordance with the Declaration of Helsinki. All participants received written information about the experiment and gave written informed consent.

All participants were male. The group with psychopathy consisted of 18 offenders, the forensic group without psychopathy consisted of 14 offenders and the control group comprised 18 healthy volunteers without criminal records or a history of psychiatric disorders who were recruited through advertisements and matched with the delinquents on age and intelligence (see Table 1 for characteristics of the study population). The Psychopathy Checklist-Revised (PCL-R; Hare, 2003) was used to assess psychopathy. The PCL-R is an instrument that allows the assessment of psychopathy through a semi structured interview and information on criminal history (Hare, 2003), and is regarded as the golden standard for the assessment of clinical psychopathy. The instrument consists of 20 items capturing behavioral correlates of core aspects of psychopathy, which are coded as either not present (0), moderately present (1), or certainly present (2). Certified psychologists administered the PCL-R after placement in the Dutch forensic mental health system and for the present study the PCL-R scores were retrieved from offenders' files. As common in European countries, participants with a score of 26 or more were included in the group with psychopathy and participants with a score below 26 were assigned to the non-psychopathy group (Hildebrand et al., 2004). As healthy controls did not have criminal records, is was not possible to obtain reliable PCL-R scores in this group. Exclusion criteria were assessed with the Dutch version of Mini International Neuropsychiatric Interview Plus 5.0.0. (Van Vliet et al., 2000) and Structured Clinical Interview for DSM-IV Axis II Personality Disorders (Weertman et al., 2000) and included all major Axis I and Axis II disorders (except antisocial personality disorder in the offender groups) or any CNS injuries (Brazil et al., 2009, 2011, 2012). Additional information was retrieved from each offender's clinical files. An estimation of intelligence level was made for all participants by using the Dutch version of the Adult Reading Test (NLV; Schmand et al., 1991). All assessments were carried out by trained psychologists.

**Table 1:** **Sociodemographic and clinical characteristics of study participants (mean [*SD*])**.

	**PP (*N* = 18)**	**Non-PP (*N* = 14)**	**HC (*N* = 18)**	***p*-value**
Age in years	42.5 (6.7)	39.7 (7.7)	37.4 (8.8)	0.15
PCL-R	31.0 (3.6)	15.8 (5.1)	n/a	0.00 ^*^

PP, offenders with psychopathy; non-PP, offenders without psychopathy; HC, healthy controls.

* significant difference between PP and non-PP.

### Design

Participants were responders in a computerized version of the modified UG with two within-subject factors: Intentionality and Context. Intentionality had two levels based on *who* selects the offer: the human player (i.e., the proposer) him/herself (intentional) or the computer (unintentional). Intentionality was thus manipulated in a binary fashion. Context had four levels based on alternatives to an unfair distribution (8:2): a fair-alternative (5:5 vs. 8:2), a hyperfair-alternative (2:8 vs. 8:2), a hyperunfair-alternative (10:0 vs. 8:2), and no-alternative (8:2 vs. 8:2). Hence, the factor Context pertains to the alternative outcome that had *not* been chosen. Pitting an unfair offer (8:2) against a fair alternative (5:5) can be seen as an explicit version of the classic UG in which decision-making is generally based on comparing any offer to a potential equal split. The resulting 8 conditions were presented 16 times each (counterbalanced for proposers' gender and position of the unfair offer). As the no-alternative condition entails an 8:2 offer for either alternative, an unfair offer (8:2) was presented in 5 of the 8 conditions, equivalent to 80 trials. The three genuine alternative offers (i.e., 5:5, 2:8, or 10:0) were selected on 48 trials, yielding 128 trials in total. Contrary to subjects' belief, all choices were computer-generated.

### Material

**Figure 1:**
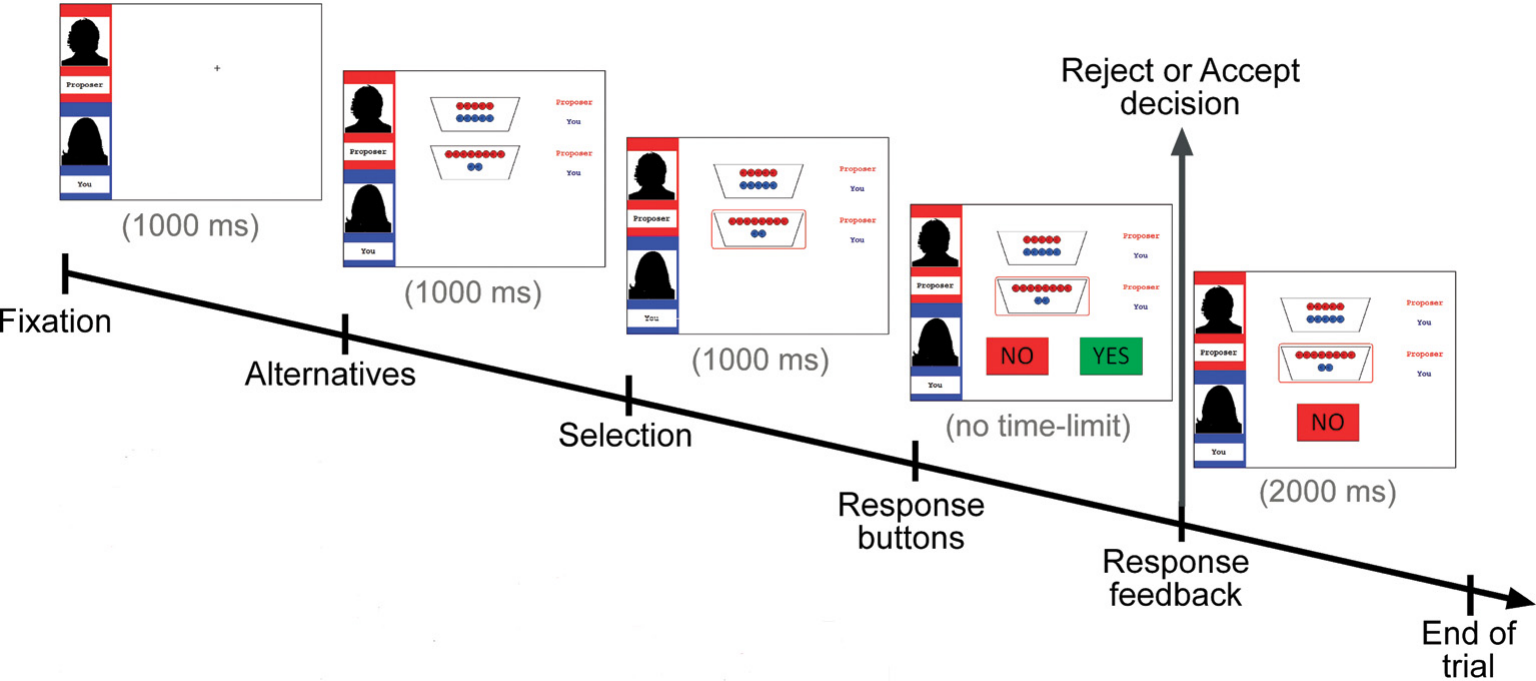
**Display of a trial in the intentional fair-alternative condition**. The name of the proposer is shown at the top (here “Proposer”) and the name of the participant is shown underneath (here “You”). The two potential distributions are specified by red and blue coins (red for proposer, blue for responder), with the offer selected by the proposer encircled in red. Participants have to indicate via button press whether to accept (“Yes”) or reject (“No”) the offer. Note that for unintentional offers (not shown here), the otherwise black silhouette of the proposer was purple with a banner displaying “Computer chooses,” which was also depicted instead of the proposer's name.

Figure 1 depicts the timeline of a trial in the intentional fair-alternative condition. Each round started with a fixation cross (1000 ms), followed by the presentation of the two available options (1000 ms). Next, the selected offer was surrounded by a red square (1000 ms). Subsequently, “Yes” and “No” buttons were presented while the selection remained visible. As the task was self-paced, participants had unlimited amount of time to respond via pressing one of two buttons using the keyboard. Participants' response remained on the screen for 2000 ms before the next round started.

### Procedure

Participants were led to believe that they were coupled with data from others who had previously participated as proposers and that they would play every trial with a new partner (Güroğlu et al., 2009; Radke et al., 2012). They were told that on some trials the other players would make an offer themselves and on other trials the computer would take over and randomly select one of the two options. Participants' task was to decide whether to accept or reject an offer. If accepted, the coins were distributed as proposed; if rejected, neither player received anything. Participants were informed that at the end of the experiment, a random number of rounds would be selected to determine their payoff. This was done to assure participants' motivation and to strengthen the concept of a one-shot game as every round could influence their financial outcome. Moreover, it was emphasized that participants' decisions also affected the other players' outcome because their payoff would be determined by participants' response, irrespective of who made the proposal in a particular round (i.e., themselves vs. computer). Proposers would be paid after all data from responders had been collected. The payoff was set around 5 Euro (± 5 cent) to manage an equal payment for all participants, but simultaneously minimize suspicion.

### Statistical analyses

Rejection rates of unfair offers were entered into a repeated measures ANOVA with Intentionality (two levels: intentional vs. unintentional) and Context (four levels: fair vs. hyperfair vs. hyperunfair vs. no alternative) as within-subject factors and Group (three levels: offenders with psychopathy, offenders without psychopathy, healthy controls) as a between-subject factor. In case of interactions involving the factor Group, separate ANOVAs for the three different groups are conducted with the above mentioned within-subject factors.

In order to test for replicating the results of Radke et al. (2012), i.e., higher rejection rates for unfair offers paired with a fair alternative when the offer was selected by proposers themselves than when selected by the computer, the effect of intentionality will be tested in the fair-alternative context on the whole group level by means of a repeated measures ANOVA with Intentionality (two levels: intentional vs. unintentional) as a within-subject factor.

## Results

There were significant main effects of Context, *F*_(3, 141)_ = 16.49, *p* < 0.001, partial η^2^ = 0.26, and Intentionality, *F*_(1, 47)_ = 4.95, *p* = 0.03, partial η^2^ = 0.10. Moreover, the interaction between Context and Group was significant, *F*_(6, 141)_ = 3.58, *p* = 0.01, partial η^2^ = 0.13. None of the interactions involving the within-subject factor Intentionality (Intentionality × Context, Intentionality × Group, Intentionality × Context × Group) was significant (all *p*s > 0.27) nor was the main effect of Group (*p* = 0.98).

Pairwise comparisons revealed that rejection rates were highest for the fair-alternative condition (63.3%) compared to the more disadvantageous alternatives (no-alternative: 41.8%, *p* = 0.001; hyperunfair: 39.1%, *p* < 0.001). Rejection rates for the hyperfair alternative condition (53.8%) were higher than for the hyperunfair and no-alternative condition (both *p*s = 0.001). The latter two conditions did not differ significantly (*p* = 1). With respect to the main effect of intentionality, rejection rates were higher when the offer was selected intentionally (52.2%) than when selected unintentionally/by the computer (49.2%).

**Figure 2:**
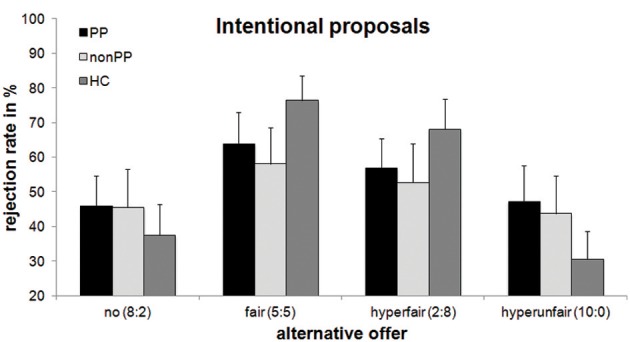
**Rejection rates of intentional unfair offers with regard to alternative offers and group**. Mean percentage and standard errors of rejection of 8:2-offers are displayed. PP, offenders with psychopathy; non-PP, offenders without psychopathy; HC, healthy controls.

**Figure 3:**
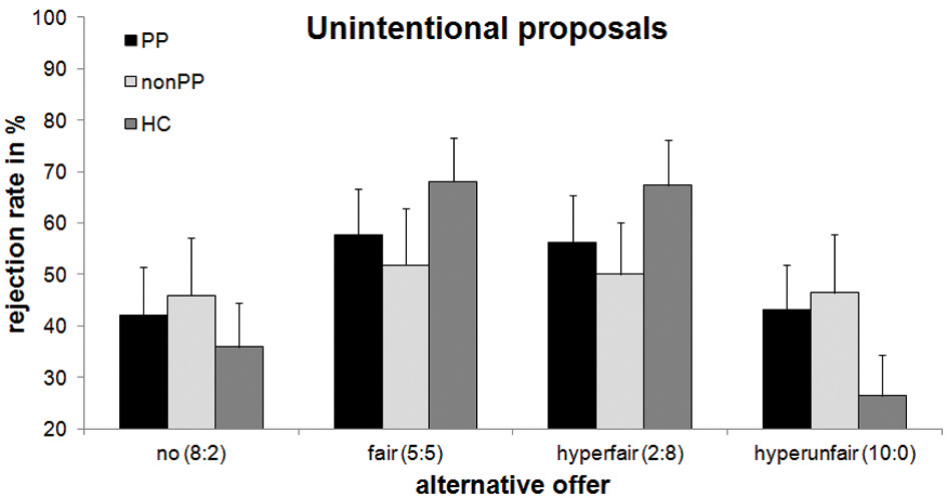
**Rejection rates of unintentional unfair offers with regard to alternative offers and group**. Mean percentage and standard errors of rejection of 8:2-offers are displayed. PP, offenders with psychopathy; non-PP, offenders without psychopathy; HC, healthy controls.

To investigate the Context × Group interaction, separate analyses for the three different groups were conducted (see Figures 2, 3). There was a significant effect of Context in healthy controls, *F*_(3, 51)_ = 14.03, *p* < 0.001, partial η^2^ = 0.45, as well as in the forensic sample with psychopathy, *F*_(3, 51)_ = 3.96, *p* = 0.039, partial η^2^ = 0.19, but not in offenders without psychopathy, *F*_(3, 39)_ = 1.63, *p* = 0.27, partial η^2^ = 0.10. For the healthy controls, the same pattern as on the whole-group level was evident: rejection rates were highest for the fair-alternative condition (72.2%) compared to the more disadvantageous alternatives (no-alternative: 36.7%, *p* < 0.001; hyperunfair: 28.5%, *p* = 0.001). Rejection rates for the hyperfair alternative condition (67.7%) were higher than for the hyperunfair and no-alternative condition (both ps < 0.002), with the latter two not differing significantly (*p* = 0.15). Reactions to the fair and hyperfair alternative conditions did not differ (*p* = 0.48). For the forensic sample with psychopathy, rejection rates were highest for the fair-alternative condition (60.7%) compared to the more disadvantageous alternatives (no-alternative: 45.1%, *p* = 0.03; hyperunfair: 43.9%, *p* = 0.02). The remaining pairwise comparisons did not yield significant differences (*p* > 0.08).

**Figure 4:**
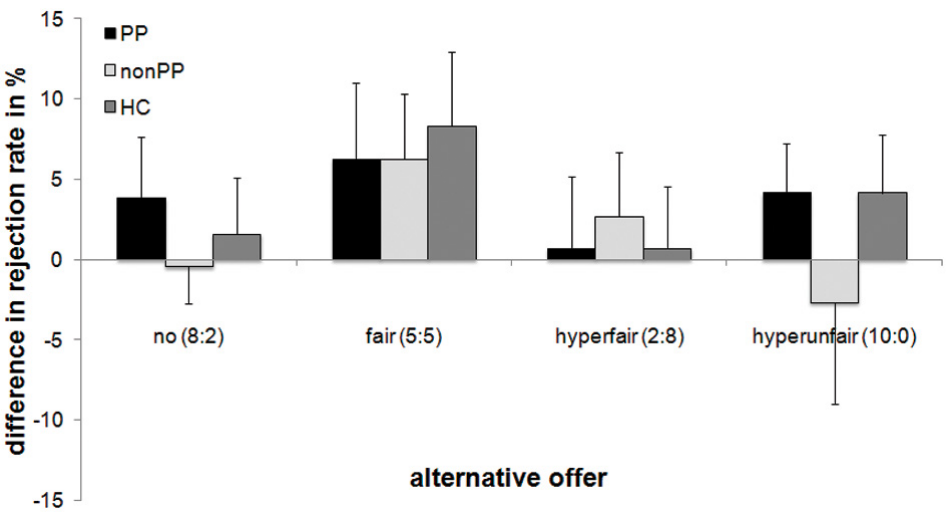
**Difference between intentional and unintentional unfair offers with regard to alternative offers and group**. Mean percentage and standard errors of rejection of 8:2-offers are displayed. PP, offenders with psychopathy; non-PP, offenders without psychopathy; HC, healthy controls.

In order to directly test for replication of Radke et al. (2012), analysis of the fair alternative condition indicated higher rejection rates for intentional (66.8%) vs. unintentional/computer offers (59.8%), *F*_(1, 47)_ = 6.78, *p* = 0.01, partial η^2^ = 0.13. In contrast, the effect of Intentionality did not reach significance for the other three contexts (all *F*s < 0.69, all *p*s > 0.41; see also Figure 4).

## Discussion

In the current study, social decision-making based on different aspects of fairness considerations was investigated in a forensic sample and a matched healthy control group. In particular, we sought to explore in how far altered moral judgments in psychopathy apply to decisions when not only moral values, but also self-relevant outcomes are at stake.

In addition to replicating previous findings on context and intentionality (Falk et al., 2003; Güroğlu et al., 2009; Radke et al., 2012), group differences in context sensitivity were evident. Essentially, offenders with psychopathy displayed a similar pattern of rejection behavior to that of healthy individuals, i.e., an effect of context. In contrast, the decisions in delinquents without psychopathy were not influenced by the alternative offer to an unfair proposal.

Recently, there has been some disagreement on which processes in fairness considerations are targeted by the context manipulation, i.e., higher-order social functions like perspective-taking (Falk et al., 2003; Güroğlu et al., 2009) or straightforward outcome comparisons (Brandts and Sola, 2001; Sandbu, 2007). This discussion (see also Radke et al., 2012) is crucial with regard to drawing inferences about possible impaired and preserved (social-) cognitive abilities in psychiatric/forensic populations. Although both processes rely on counterfactual thinking, i.e., representations of alternatives to past events (Roese, 1997), the representation of another, social agent is not necessary for comparing the outcomes for the self-resulting from the chosen and the unchosen alternative. Such a quantitative evaluation can be achieved—quite parsimoniously—without taking the perspective of another person. Our design resolves this disagreement by making the social dimension explicit, i.e., contrasting intentional and unintentional offers. The processes of outcome comparisons and intentionality considerations can thereby easily be disentangled. Importantly, the current data replicates previous findings on their relative contribution to social decision-making (Radke et al., 2012), which provides a solid basis for the investigation of intergroup variations.

As delinquents without psychopathy did not adjust their behavior to the context or intentionality of an offer, their decisions might be dominated by rather basic motives not directly targeted by these manipulations. Given that their overall mean rejection rate did not differ from the other groups either, the offenders without psychopathy seem to be guided by the magnitude of the proposal's intrinsic distribution (what the proposer gets vs. what the responder gets) with a dislike of unequal outcomes, i.e., inequity aversion. Fairness is determined by payoffs available in the here and now, which may reflect the preference for immediate options, hinting at a hyperactivity of the impulsive system (Buckholtz et al., 2010; Dean et al., 2013).

Offenders with psychopathy, on the other hand, behaved similarly to healthy volunteers. The lack of differences in overall rejection rates compared to both healthy and incarcerated individuals is in line with the behavioral results of Vieira et al. (2013), but at odds with other earlier, yet inconsistent, findings on altered responder behavior in the UG reporting higher (Koenigs et al., 2010), and lower rejection rates (Osumi and Ohira, 2010), respectively. These studies, however, also diverge in sample characteristics, with the group being either very small [*N* = 6 for high anxious psychopaths, (Koenigs et al., 2010)] or consisting of students (Osumi and Ohira, 2010) or a community sample (Vieira et al., 2013), warranting caution for generalization to a large forensic population. The main focus of the current study, however, lay not on rejection rates as such, but on its modulation by context and intentionality.

Most interestingly therefore, in the current sample, individuals with psychopathy showed an analogous sensitivity to the alternatives to a given outcome, which converges with findings on intact moral judgments (Blair et al., 1995; Cima et al., 2010; Aharoni et al., 2012), Theory of Mind (Blair et al., 1996; Richell et al., 2003) and other aspects of cognitive functioning (Blair et al., 2006; Brazil et al., 2012). Despite the behavioral similarity with healthy controls, in psychopathy the underlying mechanisms might differ and reflect a distinct motivation. For instance, in healthy individuals with psychopathic traits, rejection of unfair offers was associated with increased activation in the anterior cingulate cortex and ventromedial prefrontal cortex (Vieira et al., 2013). The recruitment of these clusters might indicate impairments in automatic emotion regulation, leading to anger-motivated instead of fairness-motivated rejection (Vieira et al., 2013).

Conversely, we observed behavioral differences in the context-dependency of rejection decisions between offenders with psychopathy compared to offenders without psychopathy. This differentiation between subgroups of violent offenders might be attributable to the cognitive nature of the task, i.e., its assessment of cognitive perspective-taking (Güroğlu et al., 2009). Previous research suggests relatively intact cognitive functioning in psychopathy, but shortages in non-psychopathy (Morgan and Lilienfeld, 2000; Gao and Raine, 2009; Brazil et al., 2012). Moreover, in contrast to emotional aspects of empathy, making inferences about others' mental states, i.e., mentalizing or cognitive inferences, does not seem to be compromised in clinical psychopathy (Blair et al., 1996; Cima et al., 2010; Shamay-Tsoory et al., 2010).

Along these lines, other (than financial) self-serving motivations supported by mentalizing might underlie the different decision patterns between offenders with psychopathy and those without. While both groups show an increased risk for frustration (Blair, 2010), individuals with psychopathy might be more successful in cognitively regulating impulsive tendencies and possibly even use a “fair” disguise instrumentally in order to obtain unobtrusive advantages, e.g., the appreciation of the experimenter. One might also speculate in how far the initial emotional responses to unfairness differ between the forensic groups. Both might react equally fervently to potentially frustrating unfair offers, but base their decisions on other features, leading to the distinct behavioral patterns. Future studies assessing physiological indicators of emotional reactivity, such as skin conductance, would be useful to explore in how far initial, affective reactions might be restrained by cognitive mechanisms of control or impression management. More tailored paradigms could also identify effects of impulsivity or serial decisions.

With regard to the relative weighting of the intention behind an action and its outcome in offenders with psychopathy (Young et al., 2012), the current data does not allow for drawing firm conclusions on the influence of intentionality for the subgroup of offenders with psychopathy. Its effects manifested only on the whole-group level, which precludes further investigation for the groups separately. Likewise, in contrast to the study by Young et al. (2012) who used hypothetical scenarios in which negative outcomes meant harm or death of another person, even the worst consequences in the current design were, naturally due to the implementation of choices, much less severe. Besides, they did not imply positive punishment, i.e., harm, but negative punishment, i.e., the withholding of coins in the case of an rejection decision and thereby forgoing potential gain. Despite the methodological strength of executing the choices in an interactive setting, this approach is less likely to trigger empathetic reactions, also since in UG settings, the most pronounced emotions arise in responders facing unfairness (Pillutla and Murnighan, 1996; Xiao and Houser, 2005).

In sum, our findings indicate discrepancies between the two offender samples: On the one hand, offenders without psychopathy seem to neglect aspects of fairness considerations that go beyond the comparisons based on payoffs, whereas, on the other hand, the decisions of offenders with psychopathy did not differ from those of healthy individuals. Distinct processes in cognition and affect might underlie these behavioral similarities. Importantly, central features of psychopathy, i.e., manipulating or deceiving others, require certain knowledge about social rules as well as cognitively taking the perspective of others, so that offenders with psychopathy might succeed in an environment where all possible outcome variants, intentions, self- and other-interests are explicitly stated. In contrast, real-life interactions with others are not only more complex and subtle, but also require emotional skills, such as generating empathic responses, regulating one's emotions, and adequately reacting to others' feelings, that are likely to be impaired in psychopathy, as evident in their antisocial lifestyles and violent crimes.

### Conflict of interest statement

The authors declare that the research was conducted in the absence of any commercial or financial relationships that could be construed as a potential conflict of interest.

## Footnotes

^1^The Pompestichting is a “TBS-clinic” located in Nijmegen. TBS is a disposal to be treated, on behalf of the state, for people who committed serious criminal offences in connection with having a mental disorder. TBS is not a punishment, but an entrustment act for mentally disordered offenders (diminished responsibility). These court orders are an alternative to either long-term imprisonment or confinement in psychiatric hospital, with the goal to strike a balance between security, treatment, and protection.
